# United States Marine Hospitals

**Published:** 1848-03

**Authors:** 


### UNITED STATES MARINE HOSPITALS.

On this subject there is an article, published in "The Boston Medical and Surgical Journal," for December 9th, 1847, which seems to be designed to show, that medical officers in charge of these establishments, and especially the officer in charge of the Marine Hospital at Chelsea, receives a salary very inadequate to the services rendered by him. This is probably true; but the amount now paid for professional services from the Marine Hospital Fund, is much greater than the Fund can bear. It is vain to attempt to augment the pay of the physicians of Marine Hospitals of the United States, until a successful effort be made to increase the revenue of the Fund, or, what would be the same in result, decrease its expenditures.

The author complains that daily newspapers of the Atlantic cities, never publish statements of the general condition of the Marine Hospital Fund. "This is the MARINE HOSPITAL FUND, paid by sailors, who never see published, for their gratification or information, the amount thus increased by their hands, for their support in time of need. By the law regulating Marine Hospitals, each sailor pays his tax of twenty cents a month, for the enlargement of this fund; and yet, *great as must be this sum* (the italics are ours) *thus accumulated, no one knows how much it is, no one knows where it goes, or whence it comes.*" The author of this communication signed, "Thirty-six years at sea," in the subsequent part of his article, speaks of "the last statistical report of Marine Hospitals," and refers to it "to show the excessive defects in a system, whose object should be the judicious expenditure of money earned with much toil, and appropriated to the relief of the sorest wants."

Had "Thirty-six years at sea" carefully examined the statistical reports of the Marine Hospital Fund, from the Treasury Department, he would have perceived, that the expenditures far exceed the receipts of the Fund, and, under existing circumstances, there is no possibility of a surplus, or accumulation. By reference to the reports from the Treasury Department on this subject, any one may know exactly, "how much" the fund "is, where it goes, or whence it comes:" and it may be also inferred that the present salaries and remuneration paid on account of the Marine Hospitals cannot be increased.

The following table, derived from public documents, shows, for a period of eleven years, the annual receipts and expenditures of the Marine Hospital Fund.

| Year. | Receipts. | Expenditures. | Excess of Expenditures. |
|---|---|---|---|
| 1836 | 67,961.02 | 89,523.94 | 21,562.92 |
| 1837 | 27,021.24 | 102,211.34 | 75,190.10 |
| 1838 | 35,234.52 | 110,291.23 | 75,056.71 |
| 1839 | 66,311.83 | 122,536.31 | 56,224.48 |
| 1840 | 71,675.91 | 136,408.64 | 64,732.73 |
| 1841 | 72,760.20 | 102,434.14 | 29,673.94 |
| 1842 | 72,429.36 | 93,531.68 | 21,102.32 |
| 1843 six months | 37,417.18 | 46,546.95 | 9,129.77 |
| 1844 | 85,017.71 | 109,541.17 | 24,523.46 |
| 1845 | 88,074.34 | 124,337.17 | 36,262.83 |
| 1846 | 88,630.60 | 87,427.23 | |
| | $712,533.91 | $1,124,789.80 | $413,459.26 |
| Deduct excess of receipts for 1846 | | | 1,203.37 |
| | | | $412,255.89 |

| | |
|---|---|
| Average annual receipts for eleven years, | $64,775.81 |
| Average annual expenditures, | $102,253.61 |
| Average annual excess of expend: over receipts, | $37,468.71 |

It will be observed, that during the past four or five years, the average annual excess of expenditures over the receipts is gradually decreasing, owing most probably to improvement in the economical administration of the fund.

It must be apparent, however, that the finances of the Marine Hospital Fund will not bear increased expenditure under any head. For the present year (1847) the receipts may be estimated at $95,000, and the expenditures at $105,000, leaving a deficit of $10,000.

It may be reasonably asked, how are these deficits made up? At this time, it is sufficient to reply, by Congressional appropriations.

"Thirty-six years at sea" asks, "How is this money expended? Its object is medical treatment; and considering the large class of persons calling for this treatment, the demand made upon the medical officers in the institutions must be very great, and every reasonable man would say they should be paid accordingly. The domestic affairs of the hospitals should by no means be neglected, but the main object of their establishment being the healing of the sick, this should be the primary aim, and this branch most liberally sustained. What is the truth on this score? By a comparison of the duties and emoluments of marine and naval hospitals, we may arrive at some knowledge of the pecuniary condition of their respective medical officers. There are in Chelsea, Massachusetts, two hospitals, the Naval and Marine, affording means for carrying out our illustration. The surgeon of the Marine Hospital has a salary of $1000 per year, without the allowance of any assistant on the part of the government; the surgeon of the Naval Hospital, if he be from ten to fifteen years standing in the service, has $2000, and the advantage of an assistant, with a salary of $950 per year; thus rendering the medical attendance of the Naval to exceed that of the Marine Hospital by $1950. Let us now compare their duties. The Naval Hospital rarely numbers more than ten patients daily, who can avail themselves of the double amount of medical attendance allowed them." The Marine Hospital for the last quarter averaged more than sixty patients daily.

From these data and these arguments, it is very clear that the physician of the Marine Hospital is not fairly remunerated for his labours. No member of the profession will say the pay is equivalent to the services rendered; but so long as the finances of the Marine Hospital Fund remain as at present, and so long too as a professional man, properly qualified, can be found ready to do the work for $1000 a year, it would be regarded as very questionable policy, to say the least, to increase the physician's salary. It is an axiom, very commonly observed in practice, never to pay more for an article or for services than is asked. Goods and services are worth what they will fetch, but no more.

The unquestionably low pay of the physicians in charge of Marine Hospitals of the United States, is not attributable so much to the Commissioner of the Marine Hospital Fund, whose aim is, or should be, to obtain as much for the money expended as possible, as to the state or condition of the profession itself. The members of it are very numerous; they are more than readily find employment; consequently, competition is very great, and it is presumed that the Commissioner of the Marine Hospital Fund, has always a choice among several *well recommended* competitors for every vacancy in Marine Hospitals. Probably these circumstances, rather than the amount of labour required, determine the rate of physicians' salaries in these institutions. It is poverty or the *res angusta domi*, which unhappily pertains to a very large proportion of graduates in medicine, which compels so many to give their services at half-price. What gentleman would give his time and labour to any institution for $1000 yearly, if he could command more for his services in private or other practice?

We repeat our opinion, that a thousand dollars a year is less than half the value of the services which a competent physician is capable of rendering, while in charge of a hospital, containing an average of sixty patients; $2500 would not be more than a fair compensation for the labour, professional and pecuniary responsibilities, of such an institution. But under existing circumstances, the Commissioner of the Marine Hospital Fund obtains medical services, as he would goods at an auction; not at their intrinsic, real worth, not at prime cost even, but for the lowest bid the auctioneer will accept. The Commissioner finds that $1000 a year is an ample bid, for the competition is so great that it commands not only medical, but in some instances also political party services in the bargain. This is not true as to medical appointments in Marine Hospitals exclusively. We find that not long since, political considerations influenced medical appointments in several of the medical charitable institutions in New York. Recently, however, the hospitals at Bellevue, Blackwell's Island, and the Lunatic Asylum, (New York,) have been placed on a different system of management. "We rejoice heartily," says a newspaper of the time, "that one of the first provisions of this new arrangement, is to exclude political considerations entirely in the choice of medical officers. Moral rectitude and professional capacity alone will be the test. In almost any profession, except medicine, a person may meddle with politics without any detriment to any one save himself; but every one knows that a political doctor is rarely overburthened with practice, as people are naturally shy of intrusting their lives to a person who spends his time in pursuits totally foreign to that profession which is to save them from death. It has hitherto been a blot on our public hospitals, that mere politicians were rewarded with stations in them, which required great professional acquirements to fill in a proper manner."

Let us return to the statement of "Thirty-six years at sea," quoted above. He exclaims, speaking of the comparative reward of the management of the domestic affairs, and medical administration of Marine Hospitals, "What is the truth on this score ?" We repeat, What is the truth ?

We believe "Thirty-Six Years at Sea" has fallen into errors, and from erroneous data has drawn incorrect inferences. He says, "the surgeon in the Naval Hospital, if he be from ten to fifteen years standing in the service, has $2000." "What is the truth on this score ?"

A surgeon in the navy of from ten to fifteen years standing, when employed on shore, receives $1750, and not $2000, as above stated. For the information of our readers we give the following statement from the pay table, published in the official Navy Register for 1844.

| | Pay per Annum. |
|---|---|
| Surgeons for the first five years standing, on shore duty, | $1250 |
| " " second five; between five and ten years, | 1500 |
| " " third five; between ten and fifteen, | 1750 |
| " " fourth five; between fifteen and twenty, | 2000 |
| " " twenty years and upwards, | 2250 |

But in order to obtain even the lowest rate of pay as surgeon, the officer will have served in the navy at least ten years as assistant; to receive the highest rate he will have been in the navy and of the profession for thirty years and upwards. It is a very long time, to look forward through thirty years—and one half of the time from a ship's deck at sea—to reach an income of $2250 annually for important professional services in a hospital. And this is the highest remuneration paid to a medical officer in charge of any hospital of the navy; at New York, Norfolk (Va.), and at Pensacola, the daily average sick list the year through is probably not less than 80. At New York recently, the sick list numbered 150 cases under treatment; and in the Naval Hospital at Chelsea, there has been as many as 70 cases.

The pay of medical officers in the navy, which by the way is smaller proportionately than that of any other grade, all things considered, is not regulated by the number of cases under treatment or the amount of responsibility, whether professional or pecuniary, but by the number of years the officer has served; so that the increase of pay is not so much a compensation for what he is actually doing, as for what he has already done, for small remuneration. There are very few competent, well educated physicians, it is hoped, who are not in the receipt of $2250 annually, after labouring in the profession for thirty years. If there be many, they have toiled to little profit, and they might have gained more, with less labour, in some other avocation.

The physicians of marine hospitals in the United States, it is believed, are generally changed with every administration of the general government at Washington. They serve no apprenticeship in the service; consequently, their remuneration cannot be well compared with that of medical officers either in the army or navy. It does not follow that because a surgeon of twenty years standing in the navy is accidentally in charge of the Naval Hospital at Chelsea, receiving $2250, that the physician of the Marine Hospital at the same place should receive more or less; nor would it follow that because a surgeon of less than five years' standing in charge of the Naval Hospital at Chelsea receives but $1250, that the physician of the Marine Hospital should not receive $2500.

Those acquainted with the subject will concur in the following opinion of "Thirty-Six Years at Sea." He says: "The whole system needs re-modelling; and by means of the present resources and the increasing demand in the country, as our ports are multiplied on every shore, lake and river, a splendid arrangement of hospitals might be made, which would be creditable to the nation, blessings to the sick sailor, and fine schools for the advancement of medical science." But this writer proposes no plan.

Having pointed out the financial condition, which seems to preclude any increase of remuneration for medical services, we propose to describe briefly the present state and system of administering the affairs of the Marine Hospital Fund, and then submit a plan which would remedy the defects, although it may not satisfy the objects of "Thirty-Six Years at Sea."

Under authority of a law of July 16th, 1798, twenty cents are monthly deducted from the wages or pay of all sea-faring people, except those employed in fishing vessels; and under the provisions of this act, all who pay the tax are entitled to "temporary relief and maintenance" in hospitals when sick or disabled. But the law does not contemplate relief or maintenance for those who may be incurable or permanently disabled; it provides only temporary relief for curable cases.

During the first few years after the institution of the Marine Hospital Fund, contracts were made, under the direction of the Treasury Department, by collectors of the customs, with city hospitals or almshouses, where they existed, and where they did not, with private individuals and physicians, for subsisting and attending such sick or disabled seamen as might claim from the collector the benefits provided by law. The nature and quality of the accommodations procured by collectors varied at different ports, and were afforded at different rates of charge, being much higher in southern than in northern ports, as a general rule: and the degree of comfort afforded to the sick sailor was as unequal as the prices paid, but never in proportion. From the manner of its financial administration—from a defective mode of collecting or disbursing the fund, or both—the expenditures from the Marine Hospital Fund were generally much greater than its receipts. It may be said to have been always in arrears; but not always so largely as the statements of the past ten or eleven years indicate, as may be seen on a preceding page.

After some years had elapsed, the plan of erecting buildings at different points, for marine hospitals, to be under the control of the Treasury Department, was begun, and appropriations were made by Congress, from time to time, to assist the Fund in executing the project.

Up to the present time, ten marine hospitals have been authorized by law. Of the whole number six are in operation, as follows: at Chelsea, Mass.; at Norfolk, Va.; at Ocracoke, N. C.; at Charleston, S. C.; at Key West, Fa.; and at Mobile, Ala.: the four others authorized are building at New Orleans; at Louisville, Ky.; at Pittsburg, Pa.; and at Cleveland, Ohio.

At Charleston, S. C., the Marine Hospital building of the United States, under an arrangement with the Treasury Department, is managed by the municipal authorities of that city, and seamen relieved at a specified rate of compensation, namely, in 1843, sixty cents daily per head, for subsistence, medical attendance, medicines, &c.; clothing and funeral charges are extra.

By the acts of July 16, 1798, and 3d of May, 1802, the Secretary of the Treasury is, *ex officio*, Commissioner of the Marine Hospital Fund; the business of it is attended by one of the clerks in his office.

Collectors of the Customs are employed as agents in collecting the fund, and making disbursements for authorized purposes; their accounts are settled by the First Auditor, and Comptroller of the Treasury. A commission of one per cent. is allowed to collectors, on disbursements from the Marine Hospital Fund.

The physicians of Marine Hospitals are appointed by the Secretary of the Treasury, and hold their office during his pleasure. The stewards and other subordinates, are appointed by Collectors, at rates of compensation subject to the approval of the Secretary of the Treasury. The hospital supplies are obtained by contract, but, it may be supposed, the arrangement places considerable patronage at the disposition of the head of the Treasury Department and of Collectors, if they be pleased to avail themselves of it for political purposes.

The physicians of Marine Hospitals receive an annual compensation of $1000; except at Norfolk, Va., and Ocracoke, N. C., where they are paid only $840; they receive neither perquisites nor emoluments of any kind.

The revenue of the Marine Hospital Fund is made up exclusively from the twenty cents deducted from the monthly pay of seamen registered for foreign commerce, and enrolled for the coasting and inland trade on the rivers and lakes.

It has been estimated that the number of persons employed as seamen and boatmen, including those engaged in the cod, whale, and other fisheries, is not less than 160,000. If all of these men were employed throughout the year, the Marine Hospital Fund would be entitled to receive from each man and boy $2 40, or an aggregate of $384,000 yearly. But the estimated receipts for 1847, amount only to $95,000, which is the sum derivable from 39,583 men and boys, employed afloat throughout the year. If there be 160,000 individuals employed afloat, it is evident, that they are under pay, on an average, less than one-fourth of their time, not three months, or the Collectors fail to obtain about three-fourths of the amount due to the Marine Hospital Fund.

It cannot be reasonably supposed that all our foreign commerce, coasting-trade, and navigation of the lakes and rivers, are conducted by so small a number of persons employed afloat as 40,000, which is about the number indicated by the receipts of the Marine Hospital Fund. Nor is it probable that sea-faring people gain a livelihood by being employed only one-fourth of their time. Is it not reasonable, therefore, to suppose, there is some defect in the system of collecting the Marine Hospital money?

From public documents* we learn that in the year 1845, the number of seamen relieved by the Marine Hospital Fund was 7918, of whom 252 died. There was expended on account of the whole, for subsistence and nursing $92,928 96; for medical services $11,148 52; for medicines $4,718 24; for clothing $422 60; for travelling expenses $328 49; for funeral expenses $891 50; for other charges (including the payment of an old claim,) $13,898 86, making an aggregate of $124,337 17. This statement enables us to understand the various purposes for which the fund is expended, and seeing that the receipts for that year were only $88,074 34, we see why that portion of the act of July 16, 1798, which directs all surplus of receipts over expenditure, to be invested in securities of the United States, becomes a dead letter.

To understand the whole subject, it is now necessary to speak of the Navy Hospital Fund, which is an entirely distinct institution.

The act of March 2, 1799, required all persons in the naval service to pay twenty cents monthly into the Marine Hospital Fund, and gives to them a right to the "same benefits and advantages" as provided for sick or disabled seamen, by the act of July 16, 1798. Although no one in the naval service ever participated in the benefits of the Marine Hospital Fund, the monthly tax continued to be paid until the 26th February, 1811, when a law was enacted instituting the Naval Hospital Fund, for the foundation of which $50,000 were drawn from the Marine Hospital Fund, being the amount estimated to have been paid by the navy into it, between the years 1799 and 1811. Under the law of Feb. 26, 1811, the Naval Asylum was erected.

* House Doc., No. 45. 1st session 29th Congress.

The sources of revenue to the Naval Hospital Fund, are the twenty cents monthly deducted from the pay of every person in the navy, and the undrawn rations of the sick, estimated at twenty cents daily per head. Other sources should be added. Interest arising from investment in securities of the United States, and all the personal property of deserters, and all balances due to dead men, should be invested on account of the Navy Hospital Fund, until claimed by legal heirs.

At present the revenue of this fund may be estimated at about $10,000 annually, and although all the current expenses of the Naval Hospitals are paid from it, the surplus on hand amounts to about $200,000. The investment of this fund was suggested in 1844, by the Hon. John Y. Mason. It has been computed that up to 1842, the loss in interest for the thirteen preceding years was $79,980 99, or an annual average loss of $6,152 38.

It is estimated that the average cost of maintaining the sick under the present system by the Marine Hospital Fund, is three dollars weekly per head.

At the Naval Hospital, New York, the daily average number of sick for the year ending June 30, 1845, was 80, and the largest number at one time was 117. The daily average cost per man for food, medicines, hospital stores, groceries, &c., was sixteen and a half cents; and including wages of all attendants, (except the pay of the surgeon and assistant surgeon,) and the cost of fuel, lights, &c., the daily average cost per man does not probably exceed thirty cents a head daily, or $2 10 per week, or about one-third less than the cost to the Marine Hospital Fund.*

With the view to equalize the expenditures and receipts of the Marine Hospital Fund, it is proposed,

*First.* To receive merchant seamen, when sick or disabled, into naval hospitals where there is no marine hospital of the United States, at a charge of thirty cents daily, to be paid from the Marine Hospital Fund.

*Second.* To place medical officers of the navy in charge of the Marine Hospitals.

*Third.* To lay a small tonnage duty on all American vessels; on

* Rep. No. 48, sick and disabled seamen. House Rep. 29th Congress, 2d session.

vessels engaged in coasting trade, say five cents per ton yearly, and on vessels employed in foreign commerce, ten cents per ton for every voyage, to be paid into the Marine Hospital Fund.

*Fourth.* A tonnage duty of five cents per ton, to be collected from foreign ships on every entry and clearance at a Custom House of the United States. In consideration of which, provision might be made for the admission of foreign seamen in our hospitals on the payment of fifty cents a day. Further, this tonnage duty, and the investing of the fund, would enable the Executive to provide permanent relief, in the naval asylums or marine hospitals, for all merchant seamen who had paid during twenty entire years, the twenty cents a month hospital money.

The first proposition is to admit seamen of the commercial marine into naval hospitals. Can this be done without interfering with the accommodations designed for seamen of the navy in naval hospitals? At Boston, or rather at Chelsea, there is a marine hospital; therefore, the naval hospital at that station might remain for the navy exclusively.

At New York there is no marine hospital of the United States; the daily average number of sick merchant seamen relieved by the marine hospital fund is, say, one hundred. They are at present accommodated in the New York City Hospital, at the rate of three dollars a week, for subsistence and medical attendance; some extra charges are also admitted. The average number of seamen of the navy, so long as the navy does not employ more than 10,000 men, in the naval hospital on that station, will not exceed eighty; therefore, the daily average aggregate would be 180. By erecting quarters for the accommodation of the medical officers on the grounds, instead of lodging them in the building, as at present, the hospital can shelter 250 patients and the necessary subordinate attendants. By making an addition of 100 to the average number of patients, a corresponding increase of attendants would not be necessary, because with an average of 50 patients, there is required a steward, matron, apothecary, two cooks, two washers, a ward-master, and four nurses. An augmentation of the number of patients to 250, would require a corresponding increase only in the number of nurses and washers, or, in round numbers, six washers and fifteen nurses would be ample for properly attending upon the whole. For an addition of four washers at $120 a year each, would be $480; four chief nurses at $180 each, would be $720, and of five assistant nurses at $120, would be $600, or an aggregate of only $1800: or, to cover all extra expenses on account of wages, say $2500, would be required. If the daily average cost be 16½ cents for subsistence and medicine with an average sick list of 80, there is no doubt it would fall to ten or twelve cents with an average list of 180—because, experience shows, that the larger the average number of patients, the smaller is the rate of expense. There is no necessary increase of the expenditure of fuel, with the increase of patients, because the building is warmed throughout, no matter whether it contains 50 or 150 patients.

At Philadelphia the average number of merchant seamen in the Pennsylvania Hospital does not exceed twenty; and the naval hospital, under the same roof with the naval asylum, will readily accommodate more than double this number in addition to the sick of the navy.

At Norfolk, Va., there is a marine hospital of the United States; therefore, the naval hospital there would not be required.

At Pensacola, there is no doubt, but the very few merchant seamen relieved under the Marine Hospital Fund, could be received into the naval hospital without inconvenience. According to the reports from the Treasury Department, the number of merchant seamen relieved at that port does not exceed two, yearly.

Consequently, the proposition does not involve the necessity of making any enlargement of the naval hospital buildings.

It may be objected that the mingling of merchant and naval seamen together when sick, would disturb the discipline of naval hospitals. This objection has little force, because the medical officer in charge of a naval hospital would necessarily possess the same powers of control over merchant seamen, as the superintendents of the civil institutions which now accommodate them. In the latter, merchant seamen find their interests best consulted by obedience to their regulations, because expulsion is the penalty of improper conduct. Seamen serve alternately, in many cases, in the navy and commercial marine.

It may be objected, that, in large cities, sick seamen constitute a large class of the patients in the city hospitals, visited by students of medicine for clinical instruction, and to withdraw them altogether in the manner proposed, would lessen the means of public instruction to the medical schools. This circumstance should operate in favour, rather than against the proposition, because, men who pay heavily, considering their resources, for being taken care of when sick, should not be forced to exhibit their misfortunes or diseases to the public gaze, in part payment for treatment, or to the profit of professors, of science, or of the institutions in which they may be placed. Paupers in this way partially pay for their accommodation in almshouses and hospitals; but seamen should not be regarded or treated as paupers when sick, simply for the reason that they are compelled to pay for proper relief and maintenance. The fact that sailors are improvident, has been made a reason for taxing them when well, in order that they should be taken care of when sick; but it surely is not just after this to impose on them a further tax on their feelings, by requiring them to exhibit their diseases in public, to benefit science or anything else. But, in fact, as far as science is interested in the question, nothing would be lost, because the medical officers of the navy employed in naval hospitals, would be improved by the enlarged experience the proposed plan would give them.

No change in the administration, or rather in the mode of disbursing the naval hospital fund, would be necessary, consequently, upon entertaining merchant seamen in naval hospitals. The charge of thirty cents a head daily would be paid by the *marine* hospital fund, and of course received by the *naval* hospital fund; the two funds would be kept distinct, as at present.

The second proposition is to place marine hospitals in charge of medical officers of the navy. A similar proposition was made by the Hon. Levi Woodbury, when Secretary of the Treasury, in a report to the Senate, dated December 11, 1837.

It may be objected, that, to carry the suggestion into practice, would require an increase in the number of surgeons. According to the Navy Register of 1847, there are sixty-nine surgeons; of this number there are forty-seven employed afloat and on shore, leaving twenty-two unemployed. Many of these are but recently from sea; and several are disabled by age or infirmity, but little doubt is entertained that of this number six surgeons can be found both competent and willing to take charge of the six marine hospitals now in operation, including that at Charleston, S. C. Of the sixty-nine surgeons, thirty-three have been commissioned as surgeons more than twelve years, and are classed with commanders in the navy. These, then, being generally men advanced in life, might be regarded by the Navy Department as exclusively eligible to serve as Fleet surgeons, and as chiefs or superintendents of naval or marine hospitals, leaving those surgeons who are classed with lieutenants in the navy, to be assigned for duty to single ships at sea, and to navy yards, rendezvous, and "receiving vessels" in the United States.

At present there are six squadrons, each requiring a surgeon of the Fleet; there are five naval hospitals, and adding six marine hospitals, there would be eleven hospitals, or eleven shore stations, (besides the Medical Bureau) to be filled; in other words, six places at sea, and twelve on shore, eighteen in all, to be occupied by thirty-three of the older surgeons.

Of the class of sloop-of-war and upwards, the force afloat in 1847, required eighteen surgeons, exclusive of surgeons of the fleet. There are, including the stations at Baltimore and Memphis, nine navy yards; five rendezvous, and four "receiving vessels," or eighteen shore stations, making the number of places afloat and ashore for surgeons of less than twelve years standing equal, or thirty-six in all.

From the above rough estimate it may be safely conjectured that no necessity to increase the number of surgeons in the navy, would arise from adding six or even ten to the present number of *shore* stations. It may be remarked also, that men who have become too infirm for active service afloat, would nevertheless cheerfully discharge the duties of a hospital surgeon, because in the latter occupation, they are not exposed to the privations and vicissitudes incident to a sea-life.

It has been objected also, by respectable medical officers in the navy, that adding the charge of marine hospitals to the present duties of Naval surgeons, would merely increase their labours without augmenting their remuneration; that those who work most industriously and skilfully enjoy no more consideration than those who are satisfied to do no more than is absolutely required in the naval service; that the proposition is calculated to outrage professional courtesy, because, if adopted, it necessarily must displace the *five* members of the medical profession who are now in charge of the marine hospitals; that marine hospitals are as well managed, professionally and financially, as naval hospitals. Of course those members of the naval medical corps who have avowed these views in a memorial, will still be opposed to any change or any improvement, if it increase their own cares and responsibilities, which is designed to benefit the whole body of merchant seamen; their sense of professional propriety is so keen, that rather than see any *five* gentlemen forced from public employment into private practice, they would forego the possibility or probability of adding to the comfort, the benefit of no less than 160,000 of their fellow citizens. In their opinions, the interests of *five* medical men are paramount to the interests of 160,000 American sailors! Is it necessary to examine the question, whether it is the interest of American sailors who pay "hospital money," to reduce the expenditures of the Marine Hospital Fund, and at the same time improve and extend sick accommodation for them? Is it, in this connexion, necessary to examine the question, whether Congress shall, year after year, appropriate from ten to thirty thousand dollars to meet deficits in the Marine Fund, in preference to passing an act which might be construed into an act of discourtesy towards *five* gentlemen, members of the medical profession? In these remarks, the writer disclaims all ideas of disparaging, in any respect whatever, the physicians of marine hopitals of the United States.

Even admitting that marine hospitals are better conducted in every respect than naval hospitals, (which may be a question not difficult to decide,) it is no reason why medical officers of the navy should not be placed in charge of marine hospitals; but it might be urged, on the contrary, as a reason why naval hospitals should be placed in charge of physicians, selected from among citizens, without any test of qualification, except that derived from introductory letters from the hands of interested friends of the same political party, no matter whether whig or democrat.

The propriety of the suggestion made cannot, and does not, rest on the result of a comparison of the management of marine and naval hospitals; or on the comparative qualifications of surgeons of naval hospitals and of physicians of marine hospitals. It is as absurd as it would be to argue that, because the physician of the marine hospital at Chelsea is a handsomer or taller man than the surgeon of the naval hospital at New York, it is manifestly improper to make medical officers in the navy eligible to the management of marine hospitals!

It is believed that the competency of surgeons of the navy to serve in marine hospitals will be conceded; that the system of examinations to which they are subjected has given an efficient body of medical men to the navy, who will compare favourably with the mass of citizen practitioners, graduates or not. The recent medical convention has sprung from the—too long continued—defective plan of manufacturing, so called, doctors; and public opinion must sustain the views expressed by that body on this important subject. To attain a thorough knowledge of the science of medicine and surgery is not an easy task; but to facilitate those who are satisfied with the name, medical schools have given their diplomas, certificates of ability, to imperfectly educated men, and boast of the number, rather than of the qualifications of their graduates. This has jeopardized the respectability of the medical profession, by bringing members into it who are not respectable because, they lack professional knowledge. "It is much easier," says a distinguished professor, "to win spurs in the barbarian contests of war, than to gain triumphs in the more glorious fields of science and of art." This truth must be evident on scrutinizing the pretensions of the great mass of practitioners, licensed and unlicensed; graduates and licenciates. The diploma has ceased to be a guarantee of qualification, and for this reason, medical men are not received into the army or navy, without careful examination.

Professor Jackson, of the University of Pennsylvania, in his introductory lecture delivered in October, 1847, holds the following language on this subject—"The students themselves will soon feel its influence (reform in medical education,) and acknowledge that it will be for their own interests. They will find that public respect and confidence, on which their success in practice depends, as public opinion becomes enlightened, will be withheld from the illiterate and uninformed, and can be obtained only by those who are well educated and thoroughly instructed. The army and navy medical boards have already shown to them, that the low standard of the medical schools does not give the requisites of a well educated practitioner; and that the diploma, conferred with little discrimination, is no evidence of qualification. The public, taught the lesson, begin to adopt the same opinions. Every practitioner now feels that it is not on his vouchers he is to rely, but that the position he occupies must depend on the evidences he gives of his intelligence, general education and proficiency in his science."

The third proposition is to impose a small tonnage duty on all American vessels, to be added to the Marine Hospital Fund; and the fourth proposition is to impose a similar duty on foreign vessels, and give their crews, in consideration of it, a right of admission when sick into our hospitals. Should such a tax be imposed, United States Marine Hospitals might be established in some of the distant ports most frequented by American ships. Such an institution at the Sandwich Islands, or at some neighbouring point, in the Pacific, would be of inestimable advantage to Americans, engaged in the whale fishery?

It may be objected, in these days of ultra "Free Trade" principles, that such a tax would be injurious to commerce; but we beg to express our most decided opposition to any dismemberment of the sentiment, which was borne aloft in the battle and on the breeze:—"Free trade and Sailors' rights." We hold that among the latter should be included a right to protection, relief and maintenance, when sick and disabled.

The Marine Hospital Fund has never been equal to the demands against it, and some means of rendering it sufficient must be devised. When Congress appropriates money to meet the deficiences of the Fund, it virtually imposes a tax, to the extent of the appropriation, on all classes of our citizens for the benefit of one class. Seamen themselves cannot be taxed more than they are at present. It seems only just, then, that those who are most immediately benefitted by their labours, should contribute something from their prolific revenues, to support the poor and destitute sailor, who has no claims on any municipality for relief. Our sailors are the life of commerce, for without them our ships must rot at our wharves instead of traversing every sea, and returning as richly freighted galeons and argosies from every clime, pouring wealth into the coffers of our merchant-princes at home. A tax of twenty cents a month, at the rate of more than two per cent per annum on their income, is imposed upon them because they are poor, improvident, and peculiarly liable to misfortune—for these reasons, this class of our fellow citizens is compelled by law to provide for its own poor. But alas! the number of poor and destitute sailors is greater than the contributions of seamen themselves can properly provide for. Therefore, let ship owners be required to contribute something towards the comfort of fellow-beings who have lost health, and risked life, while engaged in perilous labour, designed in its results to make them rich.

Next, it is suggested that all subjects, no matter what may be their nature, pertaining to nautical medicine, naval or commercial, be placed under the control of the chief of the Bureau of Medicine and Surgery in the navy department. Let him be the sole commissioner of both the naval and marine hospital funds, and place in his office the clerk of the navy hospital fund, and the clerk of the marine hospital fund, at present in the treasury department. The Collectors of customs would make the same reports touching the marine hospital fund, they do now, but instead of directing them to the Secretary of the Treasury, they would address them to the chief of the Bureau of Medicine and Surgery in the navy department, from whom they should be required to receive and execute instructions relative to sick and disabled seamen. This would relieve the secretary of the treasury of a portion of his onerous duties, and also take something from the labours of the secretary of the navy. The chief of the Medical Bureau should be then required to report annually, through the Secretary of the Navy, to Congress the con dition of both funds, showing the receipts and expenditures in detail. He would not be independent of the Secretary of the Navy, but be as much a subordinate as the Commissioner of pensions.

The following is a project of a law or bill which would effect the necessary changes, and lead to a satisfactory and systematic administration of both Hospital Funds.

It is believed that such a law would leave seamen nothing to justly complain of. But it will not answer to leave out any section of the proposed bill, because all its parts are mutually dependent, and necessary to accomplish the objects in view:—

A BILL FOR THE RELIEF OF SICK AND DISABLED SEAMEN.

*Be it enacted by the Senate and House of Representatives of the United States of America in Congress assembled*, That from and after the passage of this act, all marine hospitals of the United States, shall be, and are hereby declared to be, under the direction and management of the chief of the Bureau of Medicine and Surgery in the navy department, and the supetintendents and physicians of said marine hospitals shall be selected and appointed, from among the surgeons and assistant surgeons in the navy, in the same manner as the superintending surgeons and other officers are selected and appointed in naval hospitals of the United States.

Sec. 2. *And be it further enacted*, That Collectors of ports, in collecting districts in which naval hospitals exist, but in which there is no marine hospital of the United States, are hereby authorized and directed to grant admission to all sick and disabled seamen, who may be entitled to the benefits of the Marine Hospital Fund, into the naval hospital nearest to, or within the limits of their districts respectively; and it shall be and is the duty of the chief of the Bureau of Medicine and surgery to direct the superintendents and surgeons of said hospitals to receive all sick and disabled seamen who may present satisfactory certificates of admission from the collector of the port, and to provide for them in the same manner as sick and disabled seamen and marines are provided for in naval hospitals, according to the laws and usages of the navy.

Sec. 3. *And be it further enacted*, That a sum not exceeding thirty cents daily, shall be paid from the marine hospital fund to the navy hospital fund, for every sick or disabled seaman, admitted into any naval hospital by authority of the collectors of ports, for every day such merchant seaman may be maintained at the cost of the naval hospital fund, *provided* that clothing furnished to patients, and funeral expenses, shall not be included in the daily charge of thirty cents for maintenance and medical treatment.

Sec. 4. *And be it further enacted*, That the chief of the Bureau of Medicine and Surgery shall, in those collection districts in which there is no naval or marine hospital of the United States, provide suitable accommodations and attendance, either in private houses, or in hospitals, for such sick or disabled seamen, as may be entitled to the benefits of the marine hospital fund.

Sect. 5. *And be it further enacted*, That all American vessels employed in the coasting trade, or in the commerce of the lakes or rivers of the United States, shall pay to the marine hospital fund at a rate not exceeding five cents a ton yearly, according to their number of tons respectively; and all American vessels employed in commerce with foreign countries, shall pay to the marine hospital fund at a rate of not exceeding ten cents the ton, according to their number of tons respectively, whenever any such vessel shall be entered, and also whenever any such vessel shall be cleared at any custom house of the United States.

Sect. 6. *And be it further enacted*, Tbat any sea-faring person who may be disabled by disease, age or injury, on producing satisfactory evidence that he has paid into the marine hospital fund, or naval hospital fund, at the rate of twenty cents a month for a period not less than twenty entire years, shall be, and is hereby entitled to permanent relief and maintenance in any marine or naval asylum, or hospital of the United States, under the laws and usages of the navy.

Sect. 7. *And be it further enacted*, That all foreign vessels shall pay to the marine hospital fund, at a rate of not exceeding five cents a ton, according to their number of tons respectively, whenever any such vessel shall be entered, and also whenever any such vessel shall be cleared at any custom house of the United States, and in consideration of the payment of this duty, foreign seamen may be admitted to the temporary benefits of the marine hospital fund, *Provided*, that masters or consignees of the foreign vessel to which they belong, or the foreign consul having control or supervision of their interests, shall pay to the marine hospital fund not less than fifty cents daily for every such foreign seaman who may be admitted into any hospital of the United States, while he may be entertained in any of said hospitals.

SECT. 8. *And be it further enacted*, That the commissioner of the marine and navy hospital funds is hereby authorised to provide proper and suitable accommodations and treatment in hospitals or otherwise, for sick or disabled American seamen, in such foreign ports as he may think desirable, *Provided*, that the consent of the proper authority in such foreign port be first obtained.

SECT. 9. *And be it further enacted*, That at the expiration of one year after the death of any and all persons who have died or may hereafter die intestate, while in the navy of the United States, the Fourth Auditor of the Treasury Department shall place to the credit of the navy hospital fund all moneys or balances which may be due by the government to said deceased persons, and unclaimed by their legal heirs; and likewise all balances which may be due to deserters from the navy by the United States; and said moneys or balances shall be, and are hereby declared to be, the property of said navy hospital fund, until legal heirs shall appear. And the commissioner of the navy hospital fund is hereby authorized and directed to invest the said fund in securities of the United States, and also all moneys accruing to said fund in future, excepting so much thereof as may be required for the erection or necessary extension and repairs of hospital buildings and tenements, and the maintenance of naval asylums and hospitals for the use of the navy.

SECT. 10. *And be it further enacted*, That the chief of the Bureau of Medicine and Surgery in the Navy Department shall be, and is, the sole commissioner of the navy hospital fund, as well as of the marine hospital fund, and it shall be the duty of the said commissioner to invest all surplus moneys belonging to the marine hospital fund in securities of the United States, and also to report annually, to both houses of Congress, the condition of the navy hospital fund and of the marine hospital fund for the fiscal year next prior to the date of said report.

SECT. 11. *And be it further enacted*, That a clerk of the navy hospital fund, and a clerk of the marine hospital fund, shall be allowed in the Bureau of Medicine and Surgery, and the chief of the Bureau o Medicine is authorized to receive from the Treasury Department, and also from the Navy Department, all books, papers, &c., appertaining to the marine and navy hospital funds.

SECT. 12. *And be it further enacted*, That the commissioner of the navy and marine hospital funds is hereby authorized to receive property of any description which may be devised or bequeathed to either fund, and apply the same as the said funds are by law directed to be applied and appropriated.

SECT. 13. *And be it further enacted,* That all acts or parts of acts, contrary to the provisions of this act shall be, and are hereby repealed; *Provided,* that no treaty of amity and commerce with any foreign nation shall be affected.

According to this project of a law, marine hospitals are placed under the supervision of the Bureau of Medicine and Surgery of the Navy Department, and in charge of medical officers of the navy, under the same rules which apply to naval hospitals. The effect of the first section of the law would be, supposing that ten marine hospitals are in operation, to relieve the Marine Hospital Fund of say $1000, yearly, on account of each, or $10,000 annually. But it would require an increase of the naval appropriation, under the head of pay, to an amount not exceeding $3,500—or the difference between shore duty, and "waiting orders" of ten surgeons.

The second and third sections provide for admitting merchant seamen into naval hospitals; and the fourth, for taking care of them in private houses or hospitals, at those ports where there are no hospitals of the United States.

The fifth section provides for an increase of the revenue of the Marine Hospital Fund. It will be safe to estimate that there are 1,500,000 tons of American shipping employed in the home trade, which, at five cents the ton, would yield, say, $75,000 annually. In the year 1845, the number of tons of American shipping "cleared" for foreign commerce was 2,053,977, and the number of tons "entered" was 2,035,486 tons, or an annual aggregate of, say, 4,089,463, or, in round numbers, 4,000,000, which, at ten cents the ton, would be $400,000; or, if we say five cents the ton, $200,000. This, then, after deducting the expenses of collection, would add to the revenue of the Marine Hospital Fund about $250,000. This increase would be sufficient to carry into effect the sixth and eighth sections of the bill.

The foreign tonnage "cleared" at American custom houses in 1845, was 930,275 tons, and the number of tons "entered" was 910,563—or an aggregate of 1,840,838—or in round numbers, say 1,500,000, at five cents the ton, would yield 75,000 annually to provide for sick and disabled foreign seamen in our hospitals.

The remaining provisions of the bill seem too plain to require explanation.

At present, the revenue of the Marine Hospital Fund is so small, that the period of "temporary relief and maintenance" is limited to four months in all cases; and further, the sick list is, at some ports, restricted to a fixed number. When the sick list is full, applicants, no matter what their condition may be, must wait for vacancies, or discharges of those previously admitted into hospital. Members of the profession will perceive that in cases of phthisis, for example, that the period of limitation may be much too short; such cases, after having been in hospital four months are discharged, and become a charge on some one of the municipal charities of the port. This is equally unjust, both to patients and public.

Since its institution, the revenue of the Marine Hospital Fund has been unequal to the demands against it, and Congress has been annually called upon to make appropriations in aid of it. In a speech delivered in the House of Representatives, on the 4th February, 1847, the Hon. Daniel P. King, of Massachusetts, expressed the following views:—"My object in rising is to propose the following amendment: 'To supply deficiences in the Fund for the Relief of Sick and Disabled Seamen, $12,000.'"

"In the last year's general appropriation bill, I proposed an amendment giving twenty-five thousand dollars to supply deficiencies in the marine hospital fund. The chairman of the Committee of Ways and Means, always desirous of saving the people's money for the uses of the people's Government, strenuously opposed the measure; but the House, actuated by a sense of justice, after a full discussion, adopted the amendment, and it became a law. The same necessity for an appropriation now exists, and it shall be my endeavour to satisfy the committee that justice and good policy demand it. For more than forty years this annual appropriation has been made, and no money has been better applied or more gratefully received. Now, when by the bad policy of the Administration the revenue has been diminished, and the expenses of the Government much increased by a war which I consider wanton and unnecessary, these poor, sick, and disabled sailors must be put on half allowance, and, instead of the usual appropriation of twenty-five thousand, it is proposed, as more likely to secure the favour of the committee, to give them twelve thousand dollars. If you will allow the money to be expended where it is collected, Massachusetts sailors will ask for themselves nothing more; but with a characteristic generositv, thev desire that relief may be extended to their suffering shipmates, wherever they may chance to be overtaken by sickness and misfortune. The expense of maintenance and medical attendance is much less in the northern than in the southern hospitals. The sailors do not complain of the tax laid by the Government upon them; but they want that it should be honestly and fairly expended. If the amount of tax is not sufficient, increase it, but do not deprive them of their hard-earned rights. Individuals of them may have contributed, without complaining, for twenty or thirty years to this fund, without needing relief. When, by sickness or accident, they are now in want of its aid, it is denied them, because the fund is for the time insufficient. The amount collected in 1844 was $85,017 ; the amount expended, $109,541 ; the number of sailors relieved 7,000; of these, 720 received relief in Massachusetts, where the amount collected was $14,271; the amount expended, $11,608. You can remedy the evil by an annual appropriation; you can try the experiment, for wild experiments in legislation are the order of the day, by providing by law that no sailor shall be afflicted by sickness or accident from the first day of January to the first day of April, annually. The experiment would probably fail, but a prudent and paternal Government would not fail to make the provision contemplated by this amendment.

"By a law passed July 16th, 1798, it was provided that all masters of vessels arriving from foreign ports shall, previous to the entry of their vessels, pay to the collector of the customs a sum equal to twenty cents per month for every seaman employed on board their vessels, which sums they were authorized to retain from the wages of such seamen. Provisions nearly the same have been made in relation to persons engaged in the coasting and inland trade; and the several collectors are required to make quarterly returns to the Secretary of the Treasury ; and, from the money so collected, provision is made for the temporary relief (not exceeding four months) of sick and disabled seamen in hospitals, or such other places as may be provided.

"Seafaring men are proverbially improvident; in their generosity they would divide their last dollar with a suffering shipmate or brother tar. Many of them have no abiding home; they are citizens of the United States and have no particular claim on any municipality for relief. When overtaken by disease or poverty, they are the proper subjects of national charity. It would not be improper, then, that the needy and afflicted of their number should be provided for by the General Government. Instead of doing so you have enacted laws without their consent to compel them to provide for their own poor. This provision has been found by long experience to be insufficient. Until you have made it sufficient, do not justice and liberality require that you should supply the deficiency? and what class of our citizens are better entitled to the bounty of the Government? They brave the dangers of the ocean and the storm, that you may enjoy the luxuries of foreign climes. They are the instruments of bringing into the country the goods and merchandize, from duties on which you derive almost all your national revenues. Generous themselves, they will appreciate the generosity of the Government, and love their country all the better for its kind and not unmerited provision for them."

[Mr. K. made other statements to show the necessity and propriety of this allowance, and said he was satisfied that the House would afford this aid and comfort; and at this easy price secure the gratitude of many sailors in all portions of the country. This item was added to the bill.]

The adoption of the bill proposed, would relieve Congress from the necessity of making, annually, meagre appropriations for the relief of this Fund, and place sick and disabled seamen in a more comfortable condition than they are at present.

In conclusion, the writer begs leave to say, that he has no personal interest, direct or indirect, in the plan proposed, which must stand or fall on its own merits, after proper examination. The influence of his name can give it no weight; and as he does not seek praise for the suggestions made, he submits them honestly for the consideration of those legislators, who sincerely desire the good of our merchant sailors, now numbering, say 160,000, of our fellow citizens. He speaks for himself alone, and without asking the advice or consent of his fellow members of the medical profession.

W. S. W. R.

